# Impact of the Pro12Ala Polymorphism of the PPAR-Gamma 2 Gene on Metabolic and Clinical Characteristics in the Palestinian Type 2 Diabetic Patients

**DOI:** 10.1155/2009/874126

**Published:** 2009-10-25

**Authors:** S. Ereqat, A. Nasereddin, K. Azmi, Z. Abdeen, R. Amin

**Affiliations:** ^1^Department of Biochemistry and Molecular Biology, Medical School, Al-Quds University, East Jerusalem, Abu-Deis, P.O. Box 19356, Palestine; ^2^Al-Quds Nutrition and Health Research Institute, Al-Quds University, East Jerusalem, P.O. Box 20760, Palestine

## Abstract

Peroxisome proliferators activated receptor-gamma2 (PPAR*γ*2) represents the transcriptional master regulator of adipocyte differentiation and therefore has been suggested as a candidate gene for obesity, insulin resistance, and dyslipidemia. The objective of the study was to investigate for the first time the potential association of the most common variant Pro12Ala (p.P12A) substitution of the *PPARγ*2 gene with body mass index (BMI), blood pressure, fasting plasma glucose, plasma total cholesterol, LDL and HDL cholesterol, and plasma triglyceride in a sample of 202 (138 females and 64 male) type 2 diabetic Palestinians. Genotyping of the *PPARγ*2 p.P12A polymorphism was determined by polymerase chain reaction-restriction fragment length polymorphism (PCR-RFLP) analysis. The A12 allele was associated with lower fasting plasma glucose (*P* = .03) but had no influence on blood pressure, BMI, or other metabolic parameters. In obese patients, the p.P12A substitution was associated with elevated total plasma cholesterol levels (*P* = .02) and a tendency toward increased LDL cholesterol level (*P* = .06). In conclusion, the p.P12A variant of the *PPARγ*2 may influence cardiovascular risk through effects on lipid metabolism in obese T2D Palestinian patients.

## 1. Introduction

Type 2 diabetes mellitus (T2DM) showed to be a cocktail of genetic discovery as described by Freeman and Cox [1]. T2DM cases would have inborn with a diversity of different genetic factors that together with environmental factors combine as the primary cause and contributed to the disease.

Studies enlightening the epidemiology of diabetes mellitus among Palestinians are scarce. A Palestinian study conducted at 2001 [2] showed DM prevalence in 12% of urban Palestinian population including 492 men and women 30–65 years old. According to the WHO global estimate, and the epidemic nature of diabetes; prevalence of diabetes is expected to increase in Palestine and numbers should be revised to have more realistic estimation, which enables health providers to be aware of the disease magnitude, so that more effective health strategies can be adopted.

Preoxisome proliferator-activated receptor gamma (PPAR*γ*) is a nuclear hormone receptor which plays a critical role in regulating adipocyte differentiation and the transcription of genes that are important for lipogenesis. In macrophages, the activated PPAR*γ* promotes the cellular efflux of phospholipids and cholesterol in the form of high density lipoprotein (HDL) [3, 4]. PPAR*γ2* is a key modulator of adipogenesis and insulin signaling [5]. Cytosine to guanine single nucleotide polymorphism (rs1805192) in PPAR*γ2* gene (NM_015869) resulting in a proline to alanine substitution at codon 12, which has been found to modulate the transcriptional activity of the gene [6], and associated with altered insulin sensitivity [7]. Although most studies have shown a statistically significant T2DM risk reduction given by Ala variant [8–11], some others have not [12–15], suggesting variability in the contribution of this variant to the risk of T2DM. In a previous study of 333 Palestinian subjects (*n* = 219 T2DM patients and *n* = 114 normoglycemic controls), we were unable to demonstrate a significant association between the p.P12A variant and the T2DM among Palestinians (0.055 versus 0.048, OR = 0.87, *P* = .9) (unpublished data). The aim of this study was to investigate for the first time the relationship between p.P12A polymorphism of PPAR*γ2* gene and blood pressure, BMI and other related metabolic parameters in type 2 diabetic Palestinians.

## 2. Subjects and Methods

After an institutional review board approval and a completion of an informed consent, two hundred and two (202) diabetic type 2 patient volunteers (121 obese subjects (BMI ≥ 30 kg/m^2^) and 81 nonobese subjects (BMI < 30 kg/m^2^) were randomly recruited on the basis of routine visits to the Amari Refugee camp outpatient UNRWA clinic in the Ramallah District, 14 km north Jerusalem. The study protocol and the consent form were approved by the institutional Human Research Ethics Committee at Al-Quds University. The volunteers were unrelated, over 40 years old and diagnosed with diabetes according to WHO criteria or currently being treated with medication for diabetes. Subjects with previous diagnosis of type 1 diabetes were excluded from the study. Complete medical records including family history for diabetes, resting blood pressure, and anthropometric parameters: weight, height, and BMI (weight in kilograms divided by the square of height in meters) were obtained.

For laboratory analysis, 5 mL of blood were obtained after overnight fast. Blood samples were collected in EDTA-tubes and immediately centrifuged at room temperature. Plasma was aliquoted for glucose, cholesterol, HDL-cholesterol, and triglyceride determination. Plasma glucose was determined by a glucose oxidase method. Plasma total cholesterol, triglyceride and HDL cholesterol were determined by enzymatic methods using commercial kits (Human, Wiesbaden, Germany). Plasma LDL cholesterol was calculated by the Friedewald formula [16]. All the biochemical measures were obtained in milligram/deciliter (mg/dL), to convert plasma glucose, triglyceride, and cholesterol values into millimolar concentration (mmol/L). Multiply the given values in (mg/dL) units by 0.0555, 0.0113, and 0.0259, respectively.

Genomic DNA was extracted from peripheral blood according to standard procedures (Epicenter, Madison, Wis, USA). The p.P12A polymorphism was detected by PCR-RFLP analysis as previously described by Johansen et al. [17] with the following modifications: The PCR reactions were carried out using 100 ng of purified genomic DNA samples, with 1 *μ*M of each primer (forward/reverse) and PCR-Ready Supreme mix (Syntezza Bioscience, Jerusalem) in a final volume of 25 *μ*L.

## 3. Statistical Analysis

Statistical differences between mean values of the continuous variables of subjects with and without the polymorphism were evaluated by student's *t* test and confirmed by a nonparametric analysis using Mann-Whitney test. Chi-square analysis was performed to test the allelic frequency differences between obese and nonobese subjects. Hardy-Weinberg equilibrium was computed to the expected genotype distribution. Analysis was performed using SPSS program version 15. *P* value less than .05 was considered significant.

## 4. Results

The biochemical and anthropometric results for the 121 obese subjects and 81 nonobese subjects are shown in Table 1. No significant differences in biochemical parameters were noted between the two groups. The genotype analysis in nonobese subjects revealed that 73 individuals were homozygote for the wild P12P allele, and 8 individuals were heterozygote P12A. Among the obese group, 106 individuals were homozygote for the wild P12P allele, 14 individuals were heterozygote P12A and only one patient was homozygote A12A for the rare allele. Because there was only one homozygote patient for the rare allele, this individual was combined with the heterozygote group and compared with the homozygote wild group in all of the statistical analyses. No significant statistical correlation was observed between medication for diabetes and either genotype or gender (*P* > .05) in both groups. The overall frequency of the A12 allele in T2DM patients was 0.059 and it was in Hardy-Weinberg equilibrium. Since PPAR*γ2* is an important regulator of adipogenesis, it was relevant to verify if the p.P12A variant was associated with obesity. The frequency of the A12 allele did not differ significantly between obese and nonobese diabetic subjects (0.066 versus 0.049, *P* = 0.6). No significant differences in the genotypic distribution were found between the two groups as shown in Table 2. The presence of A12 variant was significantly associated with lower plasma glucose (174 ± 45.9 vresus 214 ± 84.9 mg/dL, *P* = .03) for the whole group (Table 3). However, there were no significant statistical differences between the PPAR*γ2* genotype and gender, age, BMI, plasma triglyceride, plasma total cholesterol, HDL and LDL cholesterol, as well as systolic and diastolic blood pressure.

Among obese subjects, p.P12A carriers showed significantly higher level of plasma total cholesterol (232.2 ± 41.8 versus 197.1 ± 52.7 mg/dL, *P* = .02) and tended with a borderline significant to have lower plasma glucose than those with P12P genotype (*P* = .05). A tendency toward increased levels of LDL cholesterol among P12A genotype were also observed but not significant (*P* = .06) (Table 3). Both subgroups showed no association of P12A genotype and triglyceride, and HDL cholesterol.

## 5. Discussion

In this study, the frequency of the A12 allele of the PPAR*γ*2 gene in diabetic subjects was similar to that reported for other Arabic populations such as Qatari and Tunisian ones [13, 14], but was slightly higher than in East Asian (Malaysians, Chinese, and Koreans) populations [15, 18] and much lower than that reported in Caucasian subjects [19]. This might be explained by the genetic background shared between our studied group and the other Arabic origin subgroups [13, 14] compared with other ethnic groups [15, 18, 19]. More comprehensive genotyping studies are needed to clarify this point. Of at most interest is to recruit and genotype large representative Palestinian T2DM patients to get conclusive results for comparison with the Caucasian group.

In agreement with early published studies, we could not demonstrate an effect of the p.P12A variant on several traits that are associated with insulin resistant syndrome, such the BMI, blood pressure, and plasma triglyceride levels [15, 20].

On the other hand, our study showed the diabetic subjects with the P12A genotype had lower fasting plasma glucose levels than those with the P12P genotype. This effect had a borderline significance in the obese group. Thamer et al. [21] indicated that the mechanism by which the A12 allele improves insulin sensitivity might involve enhanced suppression of lipid oxidation permitting more efficient glucose disposal, however, this effect should be clarified in future studies with detailed laboratory tests such as glycated hemoglobin and insulin sensitivity measures by the gold standard OGTT and/or the hyperinsulinemic euglycemic clamp [22].

Of the variables tested, the P12A genotype had the greatest effect on plasma total cholesterol with an elevated level among diabetic obese than nonobese subjects consistent with early published studies in Caucasian and Japanese populations [8, 19]. Plasma LDL cholesterol exhibited a similar trend, but did not achieve significance. Since both obese and nonobese groups had similar lipid profile (Table 1). This effect is not simply reflected by obesity, it might be revealed due to variable interactions of the Ala allele with other genetic and environmental factors. In the Finnish study, the effect of P12A genotype on T2DM risk was modified by physical activity [23]. Combining all these results together, it may suggest an intrinsic reduction in the PPAR*γ2* activity; however, it was shown that the A12 substitution in the PPAR*γ2* gene associated with reduced lipoprotein lipase activity [24] which results in impaired clearance of LDL, VLDL resulting in elevated total plasma cholesterol, LDL, and VLDL levels. In contrast, other studies did not find impact of p.P12A of PPAR*γ2* on lipid profile in diabetic and normoglycemic subjects [25]. We believe that ethnic differences, BMI, gender, physical activities, study design, and sample size all could be considered as reasons for discrepancies in association studies.

In conclusion, this study is considered to be the first to focus on molecular type-2 diabetes on Palestinians which will help in draw a baseline for further diabetes research studies in Palestine. Our findings showed no significant impact of the p.P12A PPAR*γ2* polymorphism on BMI, blood pressure and triglyceride. However, this variant in obese T2DM patients may influence cardiovascular risk through effects on lipid metabolism. The role of other polymorphisms particularly—if found—in a location near to the p.P12A is still to be defined in our population.

## Figures and Tables

**Table 1:** Anthropometrical and biochemical parameters of the study groups. Data are in means ± SD. BMI =Body mass index; SBP=systolic blood pressure; DBP=diastolic blood pressure; TC: total cholesterol; TG: Triglyceride; FPG: fasting plasma glucose; NS: not significant; D: Diet; I: insulin; IO: Insulin and oral hypoglycemic agent; O: oral hypoglycemic agent.

Parameters	Obese subjects	Nonobese subjects	*P*
	*n* = 121	*n* = 81	
Sex (M/F)	31/90	33/48	
Age (years)	58.6 + 10.1	59.9 + 10.1	NS
Age at diagnosis	49.8 + 10.1	49.6 + 10.5	NS
BMI (kg/m^2^)	35.4 + 4.9	25.8 + 2.5	<.001
SBP (mmHg)	126.1 + 18.8	130.1 + 19.0	NS
DBP (mmHg)	78.6 + 9.3	79.6 + 14.8	NS
FPG (mg/dL)	208.4 + 84.0	211.3 + 80.3	NS
TC (mg/dL)	201.5 + 52.6	189.6 + 44.3	NS
TG (mg/dL)	181.4 + 78.9	167.5 + 83.5	NS
HDL (mg/dL)	43.6 + 16.0	41.3 + 13.4	NS
LDL (mg/dL)	120.1 + 48.5	109.6 + 44.9	NS
Therapy(n) D/I/IO/O	4/22/16/79	2/22/5/52	NS

**Table 2:** PPAR*γ*2 genotype frequencies in obese and nonobese Type 2 diabetic patients. The chi-square test was performed for association.

	Genotype (n)		
	P12P	P12A	A12 frequency
Obese	106	15	0.066
Nonobese	73	8	0.049
All subjects	179	23	0.059

**Table 3:** Biochemical and anthropometrical parameters for subjects based on PPAR*γ*2 genotype. The chi-square test was performed for association.

	All subjects			Obese subjects		
	P12P	P12A	*P*	P12P	P12A	*P*
Age (years)	58.8 ± 10.0	61.4 ± 10.5	NS	58.3 + 10.4	61.3 + 7.6	NS
BMI (kg/m^2^)	31.5 ± 6.2	31.5 ± 6.7	NS	35.4 + 4.9	35.1 + 4.9	NS
FPG (mg/dL)	214.1 ± 84.9	174.3 ± 45.9	.03	214.1 ± 86.8	168.5 ± 45.5	.05
TC (mg/dL)	192.5 ± 51.0	211.1 ± 50.2	NS	197.1 ± 52.7	232.2 ± 41.8	.02
TG (mg/dL)	176.1 ± 82.2	173.6 ± 71.3	NS	181.9 ± 79.0	177.0 ± 80.6	NS
HDL (mg/dL)	42.5 ± 15.3	43.6 ± 13.5	NS	43.9 ± 16.2	41.8 ± 15.1	NS
LDL (mg/dL)	114.8 ± 47.7	124.2 ± 43.7	NS	117.0 ± 49.3	141.9 ± 37.4	.06
SBP (mmHg)	127.5 ± 18.5	129.4 ± 22.3	NS	125.7 + 18.6	128.4 + 20.6	NS
DBP (mmHg)	78.7 ± 11.6	81.0 ± 11.0	NS	78.0 + 8.9	82.8 + 11.1	NS

## References

[1] Freeman H, Cox RD (2006). Type-2 diabetes: a cocktail of genetic discovery. *Human Molecular Genetics*.

[2] Abdul-Rahim HF, Husseini A, Giacaman R, Jervell J, Bjertness E (2001). Diabetes mellitus in an urban Palestinian population: prevalence and associated factors. *Eastern Mediterranean Health Journal*.

[3] He W (2009). *PPAR*
*γ*2^Pro12Ala^ polymorphism and human health. *PPAR Research*.

[4] Cho M-C, Lee K, Paik S-G, Yoon D-Y (2008). Peroxisome proliferators-activated receptor (PPAR) modulators and metabolic disorders. *PPAR Research*.

[5] Stumvoll M, Häring H (2002). The peroxisome proliferator-activated receptor-*γ*2 Pro12Ala polymorphism. *Diabetes*.

[6] Deeb SS, Fajas L, Nemoto M (1998). A Pro12Ala substitution in *PPARγ*2 associated with decreased receptor activity, lower body mass index and improved insulin sensitivity. *Nature Genetics*.

[7] Ek J, Andersen G, Urhammer SA (2001). Studies of the Pro12Ala polymorphism of the peroxisome proliferator-activated receptor-*γ*2 (*PPAR*-*γ*2) gene in relation to insulin sensitivity among glucose tolerant Caucasians. *Diabetologia*.

[8] Mori H, Ikegami H, Kawaguchi Y (2001). The Pro12 → Ala substitution in PPAR-*γ* is associated with resistance to development of diabetes in the general population: possible involvement in impairment of insulin secretion in individuals with type 2 diabetes. *Diabetes*.

[9] Hara K, Okada T, Tobe K (2000). The Pro12Ala polymorphism in *PPARγ*2 may confer resistance to type 2 diabetes. *Biochemical and Biophysical Research Communications*.

[10] Zeggini E, Parkinson JRC, Halford S (2005). Examining the relationships between the Pro12Ala variant in *PPARG* and type 2 diabetes-related traits in UK samples. *Diabetic Medicine*.

[11] Ghoussaini M, Meyre D, Lobbens S (2005). Implication of the Pro12Ala polymorphism of the *PPAR-gamma*2 gene in type 2 diabetes and obesity in the French population. *BMC Medical Genetics*.

[12] Hansen SK, Nielsen E-MD, Ek J (2005). Analysis of separate and combined effects of common variation in *KCNJ11* and *PPARG* on risk of type 2 diabetes. *The Journal of Clinical Endocrinology & Metabolism*.

[13] Badii R, Bener A, Zirie M (2008). Lack of association between the Pro_12_Ala polymorphism of the *PPAR*-*γ*2 gene and type 2 diabetes mellitus in the Qatari consanguineous population. *Acta Diabetologica*.

[14] Bouassida KZ, Chouchane L, Jellouli K (2005). The peroxisome proliterator activated receptor*φ*
_2_ (*PPARφ*
_2_) Pro12Ala variant: lack of association with type 2 diabetes in obese and non obese Tunisian patients. *Diabetes & Metabolism*.

[15] Oh EY, Min KM, Chung JH (2000). Significance of Pro^12^Ala mutation in peroxisome proliferator-activated receptor-*γ*2 in Korean diabetic and obese subjects. *The Journal of Clinical Endocrinology & Metabolism*.

[16] Friedewald WT, Levy RI, Fredrickson DS (1972). Estimation of the concentration of low-density lipoprotein cholesterol in plasma, without use of the preparative ultracentrifuge. *Clinical Chemistry*.

[17] Johansen A, Jensen DP, Bergholdt R (2006). *IRS1*, *KCNJ11*, *PPARγ*2 and *HNF-1α*: do amino acid polymorphisms in these candidate genes support a shared aetiology between type 1 and type 2 diabetes?. *Diabetes, Obesity and Metabolism*.

[18] Tai ES, Corella D, Deurenberg-Yap M (2004). Differential effects of the C1431T and Pro12Ala *PPARγ* gene variants on plasma lipids and diabetes risk in an Asian population. *Journal of Lipid Research*.

[19] Zietz B, Barth N, Spiegel D, Schmitz G, Schölmerich J, Schäffler A (2002). Pro12Ala polymorphism in the peroxisome proliferator-activated receptor-*γ*2 (*PPARγ*2) is associated with higher levels of total cholesterol and LDL-cholesterol in male Caucasian type 2 diabetes patients. *Experimental and Clinical Endocrinology and Diabetes*.

[20] Ringel J, Engeli S, Distler A, Sharma AM (1999). Pro12Ala missense mutation of the peroxisome proliferator activated receptor *γ* and diabetes mellitus. *Biochemical and Biophysical Research Communications*.

[21] Thamer C, Haap M, Volk A (2002). Evidence for greater oxidative substrate flexibility in male carriers of the Pro 12 Ala polymorphism in PPAR*γ*2. *Hormone and Metabolic Research*.

[22] DeFronzo RA, Tobin JD, Andres R (1979). Glucose clamp technique: a method for quantifying insulin secretion and resistance. *The American Journal of Physiology*.

[23] Kilpeläinen TO, Lakka TA, Laaksonen DE (2008). SNPs in *PPARG* associate with type 2 diabetes and interact with physical activity. *Medicine and Science in Sports & Exercise*.

[24] Schneider J, Kreuzer J, Hamann A, Nawroth PP, Dugi KA (2002). The Proline 12 substitution in the peroxisome proliferator-activated receptor-*γ*2 gene is associated with lower lipoprotein lipase activity in vivo. *Diabetes*.

[25] Mancini FP, Vaccaro O, Sabatino L (1999). Pro12Ala substitution in the peroxisome proliferator-activated receptor-*γ*2 is not associated with type 2 diabetes. *Diabetes*.

